# Isoniazid and Rifampicin Produce Hepatic Fibrosis through an Oxidative Stress-Dependent Mechanism

**DOI:** 10.1155/2020/6987295

**Published:** 2020-04-23

**Authors:** Ayan Biswas, Suman Santra, Debasree Bishnu, Gopal Krishna Dhali, Abhijit Chowdhury, Amal Santra

**Affiliations:** ^1^Centre for Liver Research, School of Digestive & Liver Diseases, Institute of Post Graduate Medical Education & Research, Kolkata, India; ^2^Indiana University School of Medicine, Indianapolis, USA; ^3^JCM Centre for Liver Research and Innovations, Kolkata, India

## Abstract

**Methods:**

A combined dose of INH (50 mg) and RMP (100 mg) per kg body weight per day was administered to mice by oral gavage, 6 days a week, for 4 to 24 weeks for the assessment of liver injury, oxidative stress, and development of hepatic fibrosis, including demonstration of changes in key fibrogenesis linked pathways and mediators.

**Results:**

Progressive increase in markers of hepatic stellate cell (HSC) activation associated with changes in matrix turnover was observed between 12 and 24 weeks of INH-RMP treatment along with the elevation of liver collagen content and significant periportal fibrosis. These were associated with concurrent apoptosis of the hepatocytes, increase in hepatic cytochrome P450 2E1 (CYP2E1), NADPH oxidase (NOX) activity, and development of hepatic oxidative stress.

**Conclusions:**

INH-RMP can activate HSC through generation of NOX-mediated oxidative stress, leading to the development of liver fibrosis.

## 1. Introduction

Conclusive evidence demonstrating a cause-effect relationship between drug hepatotoxicity and development of liver fibrosis is lacking. However, several large and well-characterized drug-induced liver injury (DILI) registries, based on prolonged follow-up of well-characterized acute DILI subjects, have shown that chronic hepatitis (CH) can occur as a distinct outcome in DILI [1–5]. Persistent liver function derangements can occur in 5.7% to 18.9% of acute DILI subjects [6, 7]. In addition, histological and clinical features of CH have been observed in drug hepatotoxicity cohorts and case studies [8–10]. In most of these cases, the frequency of development of chronic DILI increases as the period of observation of the DILI cohort increases. In the absence of a biomarker for precise DILI definition as well as one indication of evolution to chronicity, the entity chronic DILI remains enigmatic despite its significance. In order to bring clarity on the issue, well-designed experimental studies are needed. In this context, looking for evidence of activation of hepatic stellate cells (HSCs) as the key player in CH and morphological proof for production of liver fibrosis by the drug is important.

Isoniazid (INH) and Rifampicin (RMP) combination therapy is one of the commonest cause to develop acute hepatotoxicity. INH is the primary toxin, and RMP potentiates its toxicity through altered kinetics of metabolites [11, 12]. Recovery from acute hepatitis, clinical or subclinical, generally occurs in clinical settings. Usually, the drugs can be continued thereafter for the originally planned duration of treatment for at least 6 months (often 9–12 months), often in a modified dosage or schedule depending on the presence or absence of liver function alterations [11–17]. Overall, INH-RMP combination treatments are associated with overt or indolent and covert hepatocyte functional changes in a significant group of exposed people and hence have the potential to cause activation of HSCs. In view of the prolonged nature of the whole process, this leads to liver fibrosis. Over and above, we have earlier demonstrated, in short term in vivo studies in BALB/c mice, that INH-RMP causes mitochondrial permeability changes and oxidative stress along with hepatocyte apoptosis [18]. Each of these has the potential to activate HSCs.

In the present study, we are seeking experimental evidence for a relationship between prolonged INH-RMP treatment and development of liver fibrosis. We describe here the findings of an in vivo study in BALB/c mice treated with INH-RMP. We wanted to address three pertinent questions in this study: (1) Can INH-RMP cause hepatic fibrosis on long-term exposure? (2) Is there any evidence for HSC activation along with associated alterations in the matrix proteins to substantiate establishment of a profibrogenic milieu on long-term INH-RMP treatment? (3) Does oxidative stress contribute to HSC activation and fibrosis on INH-RMP exposure, with an eye to get mechanistic insights in the process?

## 2. Materials and Methods

### 2.1. Animals and Their Treatment Schedule

Male mice (BALB/c; 7–8 weeks of age) were purchased from National Center for Laboratory Animal Sciences (NCLAS; Hyderabad, India). Treatment of animals and procedures performed were done in accordance with the guidelines stipulated by the animal ethics committee of the Institute of Post Graduate Medical Education & Research (I.P.G.M.E.&R.), Kolkata, India.

Mice (*n* = 24) were treated with a combined dose of INH (50 mg) and RMP (100 mg) per kg body weight per day by gavage, 6 days a week, for 4 to 24 weeks. The dosage regimen was based on our previous report [18]. Control mice received an equal volume of vehicle by gavage in the same schedule of the INH-RMP-treated mice.

### 2.2. Animal Sacrifice and Sample Collection

During the period of sacrifice, the blood was obtained by cardiac puncture and the serum samples were stored at -20°C for the measurement of alanine aminotransferase (ALT). The liver was removed, rinsed with phosphate-buffered saline (PBS), and divided into four portions: (a) fixed in 10% buffered formaldehyde (formalin) and embedded in paraffin; (b) homogenized in appropriate buffer(s) and aliquots were frozen at -70°C for biochemical assays; (c) placed in RNA later (from Ambion) solution for RNA expression study; and (d) snap frozen at -70°C for future use.

### 2.3. Serum Aminotransferases

ALT activity of serum was measured with a commercial kit (DiaSys Diagnostic Systems GmbH, Germany) according to the manufacturer's instruction.

### 2.4. Hepatic Biochemical Assays

A 10% liver homogenate was used for the determination of triglyceride (TG) content using a spectrophotometric kit from Sigma Diagnostics (St. Louis, MO, USA), levels of reduced glutathione (GSH), oxidized glutathione (GSSG), thiobarbituric acid reactive substances (TBARs), and protein content [19–22]. Activities of hepatic catalase, glutathione peroxidase (GPx), superoxide dismutase (SOD), cytochrome P450 2E1 (CYP2E1), and NADPH oxidase (NOX) were also determined [23–27]. The collagen content of the liver tissue was measured as described previously [28].

### 2.5. Histology and TUNEL Assay

Liver tissues embedded in paraffin were cut in sections (5 *μ*m) and stained with hematoxylin and eosin (H&E) and Sirius red for collagen I detection using standard procedures. Terminal deoxynucleotidyl transferase-mediated deoxyuridine triphosphate nick end labeling (TUNEL) assays were performed using the in situ cell death detection kit (Roche, Germany) according to the manufacturer's instruction. The extent of injury, apoptosis, and fibrosis was evaluated by an investigator, who was blinded to the experimental protocol and graded for steatosis by determining the overall percentage of liver parenchyma containing lipid vacuoles, with 0 = none, 1 = mild (<25%), 2 = mild to moderate (25 to <50%), 3 = marked (50 to <75%), and 4 = severe (>75%) [29]. Inflammation was graded by the presence or absence of inflammatory cells, with 0 = absent, 1 = minimal or focal occasional single clusters of inflammatory cells present in a few microscopic fields, 2 = mild inflammation, 3 = moderate inflammation, and 4 = marked inflammation [30]. The pattern of fibrosis was graded with 0 = none, 1 = portal fibrosis, 2 = periportal fibrosis or rare septa, 3 = septal fibrosis and architectural distortion but not true cirrhosis, and 4 = cirrhosis, widespread fibrosis, and hepatocyte nodule formation [31].

### 2.6. Immunostaining

Immunohistochemistry of *α*-smooth muscle actin (*α*-SMA) was performed from the paraffin-embedded sections of the liver. Briefly, deparaffinized liver sections were washed in deionized water for 1 minute and in PBS for 5 minutes, followed by permeabilization in 0.1 M citrate buffer and then blocked using PBS with 3% bovine serum albumin (BSA). The liver section was then incubated with Cy3 conjugate *α*-SMA antibody (C6198; Sigma) at 4°C overnight. After washing, the nuclei were stained with Hoechst (Sigma; 33270) for 5 minutes, washed with PBS, and mounted using Prolong Gold Antifade reagent (Invitrogen; P 36934). Slides were examined by confocal microscopy (Leica, TCS SPE; Germany).

### 2.7. RNA Isolation and Real-Time Quantitative PCR

Total RNA from liver tissue was obtained using the TRIzol Reagent (Invitrogen, Carlsbad, CA). A High-Capacity cDNA Reverse Transcription kit (Applied Biosystems) was used to generate cDNA from extracted RNA. Primers used for quantitative real-time polymerase chain reaction (qRT-PCR) are shown in Table 1. qRT-PCR was carried out on cDNA using StepOnePlus thermocycler (ABI), primer sets, and SYBR® green PCR master mix (Applied Biosystems) according to the manufacturer's instructions. Data were normalized to the expression of *β*-actin, a housekeeping gene.

### 2.8. Caspase Activity

The activity of caspase 3 was determined in liver homogenates by measuring proteolytic cleavage of the specific fluorogenic substrate DEVD-AFC (Asp-Glu-Val-Asp) (AFC: 7-amino-4-trifluoromethyl coumarin, respectively; BioVision). The results are expressed as the percentage of control.

### 2.9. Cytokine Quantification

Hepatic transforming growth factor *β*-1 (TGF-*β*1) levels were evaluated by enzyme-linked immunosorbent assay (ELISA) using quantikine kits of R&D system.

### 2.10. Western Blotting

For western blotting, tissue lysate was prepared using RIPA buffer (Cell signaling Technology, USA) with protease inhibitors cocktail (Roche Diagnostics, Mannheim, Germany). The lysates were centrifuged at 14,000 × g for 20 min at 4°C. The protein content of the supernatant was determined with the Bradford protein assay (Sigma, USA). Forty micrograms protein was resolved on sodium dodecyl sulfate–polyacrylamide gel electrophoresis (SDS-PAGE) after denaturing in sample buffer and transferred onto polyvinylidene fluoride membranes (PVDF; Thermo Fisher Scientific, USA). After blocking with 5% BSA, the blots were probed with the following antibodies: mouse monoclonal *α*-SMA (1 : 300; Santa Cruz Biotechnology), mouse monoclonal beta actin (1 : 1000; Santa Cruz Biotechnology), mouse monoclonal collagen 1A1 (1 : 300; Santa Cruz Biotechnology), mouse monoclonal anti Bax (1 : 300; Santa Cruz Biotechnology), mouse monoclonal Bcl2 (1 : 300; Santa Cruz Biotechnology), and mouse monoclonal cytochrome c (1 : 300; Santa Cruz Biotechnology). The immune complexes were visualized using the enhanced chemiluminescence (ECL) method.

### 2.11. Statistical Analysis

Results are expressed as the mean ± SD. Student's *t*-test was used to evaluate statistical differences between groups, and the Mann-Whitney test was used for the comparison of histological findings. A *p* value less than 0.05 was considered significant.

## 3. Results

### 3.1. Oxidative Stress and Liver Injury

Liver injury due to prolonged INH and RMP cotreatment in mice was assessed by measuring serum ALT level as well as histological evaluation of liver specimens. We observed a trend towards increase in serum ALT level at 4, 12, and 24 weeks of INH and RMP cotreatment (52.80 ± 3.91 IU/L at 4 weeks of treatment compared to 26.40 ± 5.41 IU/L in control mice, *p* < 0.01; 63.75 ± 4.45 IU/L at 12 weeks of treatment vs. 26.60 ± 2.26 IU/L in control mice, *p* < 0.001; and 55.50 ± 5.50 IU/L at 24 weeks of INH and RMP cotreatment against control 24.66 ± 1.52 IU/L, *p* < 0.001). We therefore assessed activities of hepatic antioxidant enzymes like SOD and other GSH-related enzymes in mice cotreated with INH and RMP. Following 4 weeks of INH-RMP treatment, activities of GPx and catalase were significantly increased (Table 2). From 12 weeks and onwards of cotreatment of INH and RMP to mice, the activities of hepatic SOD, GPx, and catalase were significantly decreased (Table 2), indicating persistent oxidative stress in the liver.

### 3.2. Prolonged INH-RMP Treatment Causes Hepatic Fibrosis

**Figure 1:**
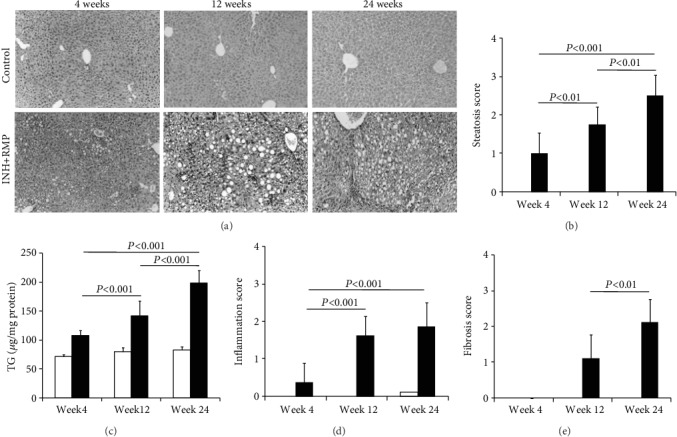
INH and RMP induced chronic liver injury in mice. Paraffin-embedded liver sections were stained with H&E, examined blindly, and graded for steatosis, inflammation, and fibrosis as described in Materials and Method. (a) H&E staining of control and INH and RMP treatment mice for 4, 12, and 24 weeks, respectively. Magnification: 10x. (b) Hepatic steatosis score, (c) liver triglyceride content of the control and experimental groups, (d) inflammation score in the liver in different weeks after INH-RMP treatment, and (e) fibrosis score are depicted as the mean ± SD of eight mice per group. White bars are for control while black bars are for INH+RMP-treated mice.

Histological changes in the liver due to prolonged INH-RMP treatment included fat infiltration, necrosis, inflammation, and, most importantly, hepatic fibrosis. Steatosis was pronounced all through the 24 weeks of exposure (Figures 1(a) and 1(b)).

Liver TG levels increased also on INH-RMP treatment (Figure 1(c)) in a temporal sequence that paralleled hepatic steatosis. Minimal inflammation and absence of fibrosis were observed at 4 weeks, whereas mild to moderate inflammation and mild portal fibrosis in the liver were evident at 12 weeks (Figures 1(d) and 1(e)). There was a progressive increase in the inflammatory cell infiltration, and the extent of periportal fibrosis was observed in the liver of mice treated with INH-RMP at 24 weeks (Figures 1(d) and 1(e)).

**Figure 2:**
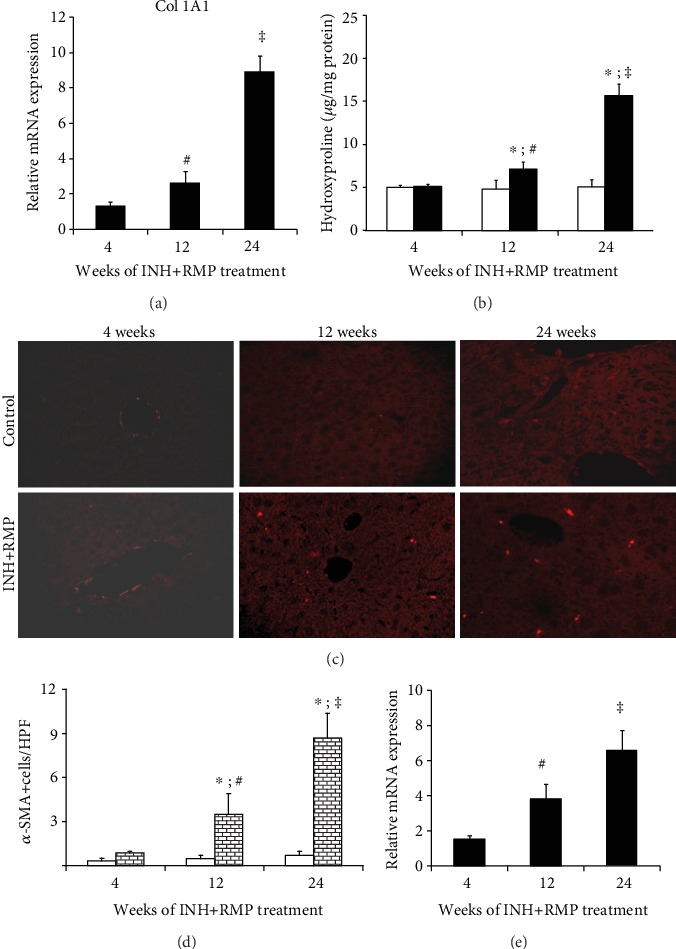
INH and RMP induced HSC and collagenesis. Activation of stellate cells and increased hepatic collagenesis at different time periods after INH and RMP treatment. (a) mRNA expression of Col1A1 in the liver at different time points (4-24 weeks) of INH and RMP treatment. (b) Hepatic hydroxyproline content of INH and RMP-treated mice at different time periods. White bars are for control while black bars are for INH+RMP-treated mice. (c) Confocal laser scanning fluorescence microscopy of *α*-SMA demonstrates the localization of activated HSCs in a mouse liver. Magnification: 40x. (d) The numbers of activated HSCs as identified by the immunohistochemistry of *α*-SMA from the paraffin sections of the liver tissues are shown graphically. 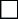 is the control, and 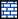 is the INH+RMP-treated mouse. (e) mRNA expression of *α*-SMA in the liver at 4, 12, and 24 weeks of INH and RMP treatment. The results are expressed as the mean ± SD of 8 mice per group. (^∗^*p* < 0.05 versus the vehicle-treated control group; ^**#**^*p* < 0.05 versus INH and RMP-treated mice for 4 weeks; ^**‡**^*p* < 0.05 versus INH and RMP-treated mice for 4 and 12 weeks in (a), (b), (d), and (e)).

INH-RMP treatment induced collagen 1A1 (COL1A1) mRNA (Figure 2(a)) expressions at 12 weeks, which showed further increase at 24 weeks (Figure 2(a)). Changes in hepatic hydroxyproline content, an amino acid specially contained in collagen, paralleled the induction of COL1A1 mRNA expressions, at different time periods of INH and RMP cotreatment (Figure 2(b)).

### 3.3. INH-RMP Treatment Is Associated with Stellate Cell Activation and Matrix Remodeling

We examined the number of activated HSCs by immunohistochemistry of *α*-SMA using a confocal microscope at different time periods of INH-RMP treatment to seek evidence for activation of HSCs (Figure 2(c)).

The number of activated HSCs progressively increased over time and showed a relationship with the duration of INH-RMP treatment in mice (Figure 2(d)). In addition, we observed an increase in *α*-SMA mRNA expression in the liver tissue, beginning at 12 weeks of INH-RMP treatment (Figure 2(e)).

**Figure 3:**
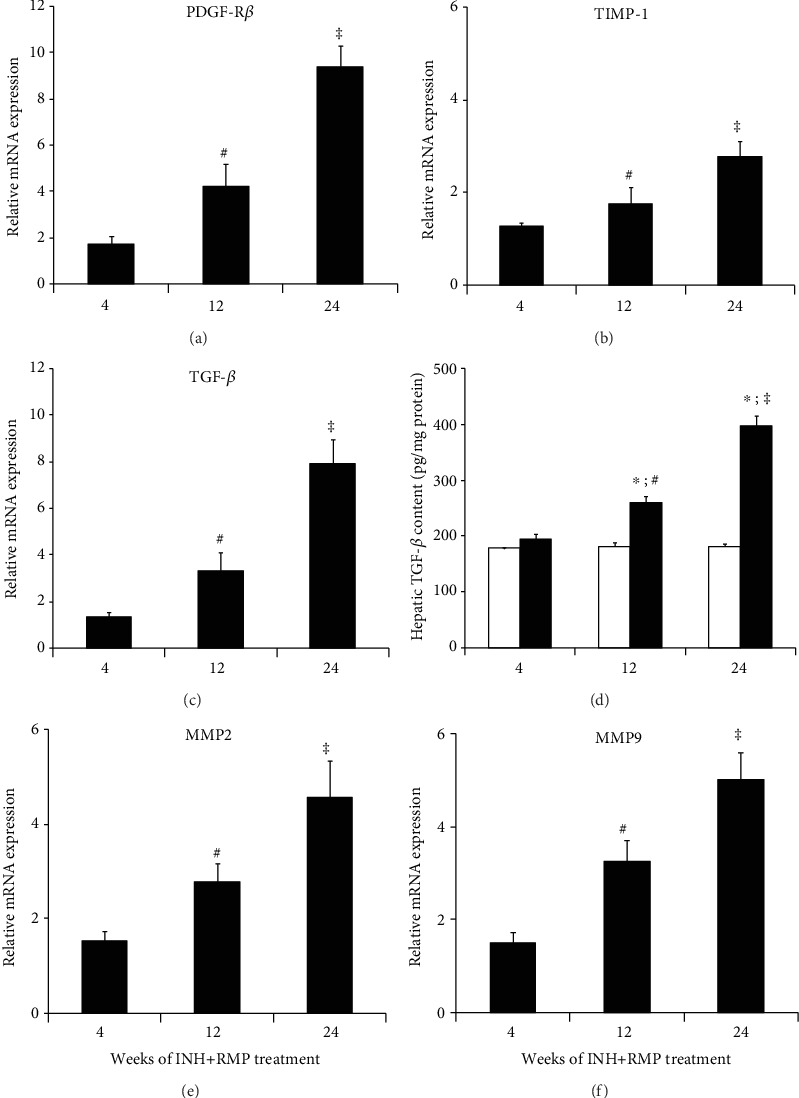
INH-RMP treatment and liver fibrosis in mice. Quantitative RT-PCR demonstrates enhanced expression of (a) PDGF-R*β*, (b) TIMP-1, (c) TGF-*β*, (e) MMP2, and (f) MMP9 in liver tissues during long-term INH-RMP treatment. ^**#**^*p* < 0.05 versus INH and RMP-treated mice for 4 weeks; ^**‡**^*p* < 0.05 versus INH and RMP-treated mice for 4 and 12 weeks. (d) Hepatic TGF-*β* protein level of mice treated with INH-RMP for different time periods. Hepatic TGF-*β* content was quantified by ELISA. The results were expressed as the mean ± SD of 8 mice per group. ^∗^*p* < 0.05 versus the vehicle-treated control group; ^**#**^*p* < 0.05 versus INH and RMP-treated mice for 4 weeks; ^**‡**^*p* < 0.05 versus INH and RMP-treated mice for 4 and 12 weeks. White bar indicates only vehicle-treated control mice, while black bar indicates INH-RMP-treated mice.

Proliferation and activation status of HSCs were assessed through expression of candidate molecule platelet-derived growth factor receptor *β* (PDGF-R*β*). As shown in Figure 3(a), PDGF-R*β* mRNA expression showed incremental increase after 12 and 24 weeks, consistent with the activation of HSCs.

Next, we assessed the tissue inhibitor of matrix metalloproteinase-1 (TIMP-1) expression in the liver tissues of mice and observed the induction of TIMP-1 mRNA after 12 and 24 weeks of INH-RMP treatment (Figure 3(b)). TIMP-1 is synthesized and secreted by activated HSCs in response to fibrogenic cytokines, in particular to transforming growth factor *β*1 (TGF-*β*1) [32]. There was a significant increase in hepatic TGF-*β* protein levels after 24 weeks of INH-RMP treatment compared to control mice (Figure 3(d)), a finding that was also confirmed by mRNA expression for TGF-*β*1 (Figure 3(c)).

Finally, we also assessed mRNA expression of matrix metalloproteinases 2 and 9 (MMP2 and MMP9), matrix remodeling-associated molecules [33] which showed marked upregulation between 12 and 24 weeks of INH-RMP treatment (Figures 3(e) and 3(f)).

### 3.4. Increased Oxidative Stress Is Related with Hepatic Fibrosis in Long-Term INH-RMP Treatment

During metabolism of INH-RMP, significant stress is being generated within the hepatocytes by the formation of reactive oxygen species (ROS) which is a potential mediator of HSC activation.

**Figure 4:**
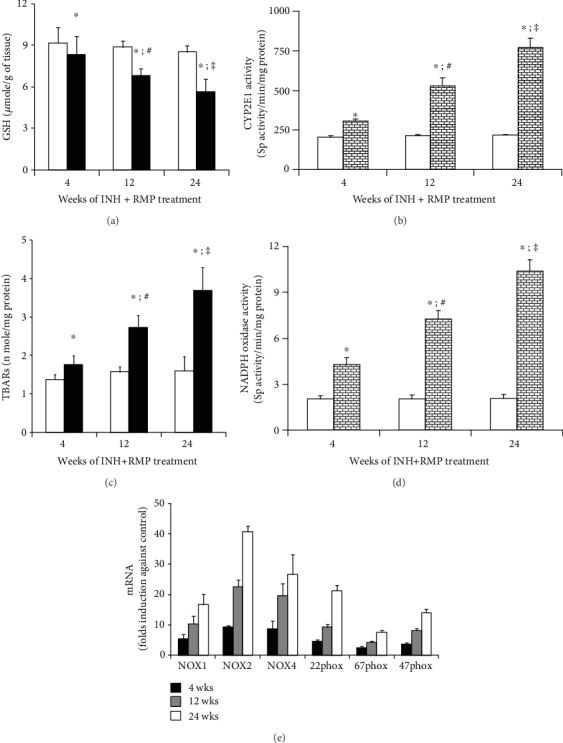
INH and RMP treatment and hepatic oxidative stress. (a) Hepatic GSH and (c) TBAR content in control (white bar) and INH+RMP-treated (black bar) mouse at different weeks. Loss of hepatic GSH content (a) and increased TBAR content (c) in the liver was used as a marker of hepatic oxidative stress. (b) Hepatic CYP2E1 activity in mice. (d) Hepatic NOX activity in mice, for (b) and (d). 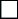 is the control, and 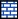 is the INH+RMP-treated mouse; (e) mRNA expression of different isoforms of NOX. Data (a–e) are the means ± SD of 8 mice per group (^∗^*p* < 0.05 versus vehicle-treated control group; ^**#**^*p* < 0.05 versus INH and RMP-treated mice for 4 weeks; ^**‡**^*p* < 0.05 versus INH and RMP-treated mice for 4 and 12 weeks in (a)–(d)).

We evaluated the oxidative stress markers that revealed a significant decrease in hepatic GSH level (Figure 4(a)) and an increase of lipid peroxidation as evident by thiobarbituric acid reactive substance (TBAR) level (Figure 4(c)). All these data suggested the development of oxidative stress during INH-RMP treatment.

In view of the critical role of CYP2E1 in oxidative stress in INH-mediated hepatotoxicity, we measured hepatic CYP2E1 activity. This showed progressive increase from 4 to 24 weeks of INH-RMP treatment (Figure 4(b)).

Parallel to CYP2E1 activity, NOX activity increased progressively with the duration of INH-RMP treatment (Figure 4(d)). To confirm further that INH-RMP activates NOX in a murine liver, a real-time mRNA expression study of the NOX subunits was performed which revealed a significant increase in the expression of different subunits (Figure 4(e)).

### 3.5. INH-RMP Treatment Increases Apoptosis of Hepatocytes in a Manner Chronologically Relevant to Hepatic Fibrosis

**Figure 5:**
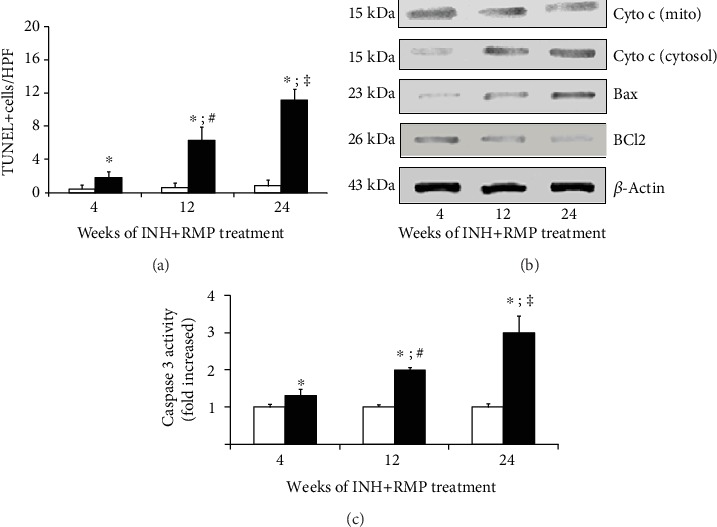
Long-term INH-RMP treatment increases apoptosis of the hepatocytes in mice. (a) Apoptosis in the liver sections was assessed by TUNEL assay. Eight different fields from each liver section were observed, and the mean value was plotted graphically. (b) Western blot analysis for cytochrome c, Bax, and Bcl2 from liver extracts from INH-RMP-treated mice at the end of 4, 12, and 24 weeks. (c) Caspase 3 activity of liver extract was determined using a fluorometric assay with Ac-DEVD AFC, in control and INH and RMP-treated mice after 4, 12, and 24 weeks. Results are presented as the fold increase from control values. The results of (a) and (c) were expressed as the mean ± SD of 8 mice per group (^∗^*p* < 0.05 versus the vehicle-treated control group; ^**#**^*p* < 0.05 versus INH and RMP-treated mice for 4 weeks; ^**‡**^*p* < 0.05 versus INH and RMP-treated for 4 and 12 weeks). White bar indicates only vehicle-treated control mice, while black bar indicates INH-RMP-treated mice.

In the current context of long-term exposure to INH-RMP, we studied the incidence of apoptosis in the liver tissue. The number of apoptotic cells showed a gradual increase with INH-RMP treatment from 12 to 24 weeks, expressed as an increase in the percentage of TUNEL positive nuclei (Figure 5(a)). Next, we observed a time-dependent decrease in the expression of the specific antiapoptotic protein Bcl-2 (Figure 5(b)), which has been shown to act on the mitochondria and prevent the release of cytochrome c and subsequent caspase activation [34].

As illustrated in Figure 5(b), a progressive increase in translocation of cytochrome c in the cytosol and increased proapoptotic Bax expression at different time points of INH-RMP treatment by western blot were consistent with the findings from the TUNEL assay. To confirm these findings, caspase 3 activity was estimated in the cytosolic fraction of the mouse liver and a progressive increase was revealed in INH-RMP-treated mice from 12 to 24 weeks (Figure 5(c)) compared to control mice.

The increased expression of proapoptotic Bax molecules strongly suggests that INH-RMP treatment causes cell death mediated by the mitochondrial apoptotic pathways.

## 4. Discussion

We show here that INH-RMP treatment in long term in a mouse model can lead to HSC activation and liver fibrosis, acting through liver cell injury mechanisms that involve NOX-dependent oxidative stress and apoptosis of hepatocytes.

Our experiments provide evidence in support of the emerging clinical data for chronic DILI [1–7]. INH caused 2.7% of the chronic DILI in the DILIN data base and is an important component of CH-producing agents in DILI [6, 35]. In this study, we raised two research questions: (a) can INH-RMP produce hepatic fibrosis on long-term exposure, as is commonly used in clinical practice? (b) How can INH-RMP connect with the liver cell injury-repair mechanisms, including oxidative stress, apoptosis of hepatocytes, and HSC activation pathways in producing liver fibrosis?

Given the exploratory nature of the primary research question, we designed in vivo mouse experiments of long-term INH-RMP exposure to seek relevant morphological and functional evidence in this respect. This approach provides robust data to suggest existence of a profibrogenic state in the liver on long-term exposure to INH-RMP.

In the in vivo study, we used INH-RMP combinations in order to capture the real life scenario in antitubercular therapy where they are used together for at least 6 months. INH-RMP combination therapy is the most common cause of acute DILI and drug-induced acute liver failure in India [36, 37]. Of the two, INH is the primary hepatotoxic drug in such combinations and RMP modifies the kinetics of toxic metabolite generation through its ability to induce microsomal enzymes. RMP, therefore, primarily plays a role in potentiating INH hepatotoxicity [12, 13, 18]. The doses of INH and RMP used in the present study are about 10 times the human doses on a milligram per kilogram basis; however, they may be equivalent to the human dose but on a body surface area basis [18].

An intriguing aspect of the histology was macrovesicular steatosis along with inflammatory cell infiltration in the early stages of INH-RMP exposure even when fibrosis has already appeared. We have also observed pericellular fibrosis on prolonged therapy—the fibrosis pattern that correlates with steatohepatitis. Histology in INH-RMP hepatotoxicity has been assessed mostly in the setting of acute liver failure and is characterized by varying amounts of necrosis and inflammation. Prominent steatosis has been observed in some human studies of nonliver failure hepatotoxicity [38, 39]. Previously, we have demonstrated that INH-RMP combination causes acute hepatotoxicity through mitochondrial dysfunction, steatosis, and hepatocyte apoptosis [18]. Progressive increase of cell death and inflammation in the liver as observed in the present study are the characteristic features that are associated with chronic liver injury leading to the progression of the development of fibrosis. Cell death is the primary precipitating event that triggers activation of inflammatory and fibrogenic signals. In the current experiments, we observed CYP2E1-dependent and NOX-mediated oxidative stress along with apoptosis increasing linearly over a period of prolonged exposure. Oxidant stress stimulates apoptosis, and we could document an increase in caspase 3, cytoplasmic translocation of cytochrome c, and reciprocal expressions of the proapoptotic Bax and the antiapoptotic BCl2 proteins in the current study, indicating the previously described mitochondrial pathways of apoptosis to be active even during the prolonged therapy periods. Further, we found increased expression of NOX that produces ROS and stimulates HSCs, over the entire duration of experiments, suggesting a nonmitochondrial pathway of oxidative stress generation also to be active. Apart from the conventional phagocytic NOX2, the nonphagocytic NOX4 was progressively expressed in the liver due to INH-RMP treatment.

In the context of HSC activation, it is important that both oxidative stress and hepatocyte apoptosis are potent mitogens for HSCs [40–42]. We, interestingly, found a steady time sequence relationship of the events that led to liver fibrosis. In the present study, we have seen the activation of the HSCs that depend largely on oxidative stress. Liver fibrosis results from deposition of type I collagen with simultaneous inhibition of its degradation. In the present study, we observed increased TIMP1 mRNA expression in the liver due to prolonged exposure of INH-RMP treatment in mice. TIMP1 is synthesized and secreted by activated HSCs under the influence of TGF-*β*1, which is also increased in the liver of mice due to INH-RMP treatment in the present study. Thus, progressive building up of oxidative stress over time was correlated with expression of HSC activation and proliferation markers starting 12 weeks of exposure of INH-RMP. This, along with activation of remodeling matrix (MMP2, MMP9, and TIMP1) and increasing COL1A1 mRNA expressions and collagen content increments, and most importantly, periportal fibrosis evident on histology was at maximum expressions at 24 weeks.

The strength of the present study is in the robustness as well as the novelty of the datasets, with the in vivo designs pursued to address the primary question of HSC activations and fibrogenesis in chronic drug toxicity. Additionally, the ability to demonstrate with precision the relevant pathophysiological changes in a sequential manner beginning with cell injury and finally the pathways that mediate the changes described are all too convincing. We believe this to be the first detailed morphological, functional description of development liver fibrosis, the critical component in CH, in the setting of DILI, and the connotations of the findings are fairly wide.

In conclusion, we have been able to demonstrate that prolonged therapy with INH-RMP can lead to HSC activation and liver fibrosis in a mechanism that is dependent on oxidative stress. Our study provides initial experimental evidence to a simmering body of clinical data suggesting drugs to be important agents in CH.

## Figures and Tables

**Table 1:** PCR primers.

Primer		Primer sequence
NOX1	Forward	5′-CTGACAAGTACTATTACACGAGA-3′
NOX1	Reverse	5′-CATATATGCCACCAGATTAGGGA-3′
NOX2	Forward	5′-CTTTCTCAGGGGTTCCAGTG-3′
NOX2	Reverse	5′-TCTTCCAAACTCTCCGCAGT-3′
NOX4	Forward	5′-TGAGGAGTCACTGAACTA-3′
NOX4	Reverse	5′-TGACTGAGGTACAGCTGGA-3′
p22^phox^	Forward	5′-TGGACGTTTCACACAGTGGT-3′
p22^phox^	Reverse	5′-TAGGCTCAATGGGAGTCCAC-3′
p47^phox^	Forward	5′-CACCGAGATCTACGAGTTCCA-3′
p47^phox^	Reverse	5′-TGTCAAGGGGCTCCAGATAG-3′
p67^phox^	Forward	5′-GCCACAGTCATGTTCAATGG-3′
p67^phox^	Reverse	5′-ACAAAAGCCTTCGGGAAAAT-3′
PDGF-R*β*	Forward	5′-AGCACCTACCTGCCTCTGAA-3′
PDGF-R*β*	Reverse	5′-GCACGGCAGTGTAGAGAACA-3′
*α*-SMA	Forward	5′-ACTACTGCCGAGCGTGAGAT-3′
*α*-SMA	Reverse	5′-AAGGTAGACAGCGAAGCCAA-3′
TGF-*β*	Forward	5′-GTCAGACATTCGGGAAGCAG-3′
TGF-*β*	Reverse	5′-GCGTATCAGTGGGGGTCA-3′
COL1A1	Forward	5′-GAAACCCGAGGTATGCTTGA-3′
COL1A1	Reverse	5′-GACCAGGAGGACCAGGAAGT-3′
TIMP-1	Forward	5′-ATCAATGCCTGCAGCTTCTT-3′
TIMP-1	Reverse	5′-ATCTCCAAGTGCACAAGCCT-3′
MMP2	Forward	5′-GAGAAGGATGGCAAGTATGGC-3′
MMP2	Reverse	5′-GCGGTCTCGGGACAGAATCC-3′
MMP9	Forward	5′-GAGTGGACGCGACCGTAGTTGG-3′
MMP9	Reverse	5′-GTACATGAGCGCTTCCGGCACGC-3′
*β*-Actin	Forward	5′-TGGAATCCTGTGGCATCCATGAAAC-3′
*β*-Actin	Reverse	5′-TAAAACGCAGCTCAGTAACAGTCCG-3′

NOX = NADPH oxidase; PDGF-R*β* = platelet-derived growth factor receptor *β*; *α*-SMA = *α*-smooth muscle actin; TGF-*β* = transforming growth factor *β*; COL1A1 = collagen 1A1; TIMP = tissue inhibitor of matrix metalloproteinase; MMP = matrix metalloproteinase.

**Table 2:** INH and RMP cotreatment and hepatic oxidative stress.

Parameters	Groups					
	Cont_4_	INH-RMP_4_	Cont_12_	INH-RMP_12_	Cont_24_	INH-RMP_24_
Cytosolic fraction (*n* = 8)						
SOD (Sp activity/min/mg protein)	125.83 ± 5.48	142.18 ± 23.13^∗^	123.46 ± 7.14	72.87±17.02^∗,#^	121.04 ± 11.61	55.06±10.74^∗,‡^
GPx (Sp activity/min/mg protein)	6.44 ± 0.66	7.32 ± 0.34^∗^	6.24 ± 0.59	4.61±0.34^∗,#^	6.86 ± 0.62	2.56±0.22^∗,‡^
Catalase (Sp activity/min/mg protein)	5.93 ± 0.54	6.51 ± 0.31^∗^	6.06 ± 0.39	4.65±0.38^∗,#^	6.41 ± 0.66	2.67±0.36^∗,‡^

Mice were cotreated with 50 mg INH and 100 mg RMP/kg body weight by oral gavage for 6 days a week for 4, 12, and 24 weeks, respectively, and were subsequently sacrificed. The results are expressed as the mean ± SD of 8 mice in each group. ^∗^*p* < 0.05 vs. respective control; ^#^*p* < 0.05 vs. INH-RMP treatment for 4 weeks; ^‡^*p* < 0.05 vs. INH-RMP treatment for 4 and 12 weeks. Abbreviations: Cont_4_, Cont_12_, and Cont_24_ are respective controls of 4, 12, and 24 weeks while INH-RMP_4_, INH-RMP_12_, and INH-RMP_24_ are INH-RMP-treated animals for 4, 12, and 24 weeks, respectively.

## Data Availability

The data used to support the findings of this study are included within the article.

## Abbreviations

CH: Chronic hepatitis
DILI: Drug-induced liver injury
INH: Isoniazid
RMP: Rifampicin
HSCs: Hepatic stellate cells
CYP2E1: Cytochrome P450 2E1
NOX: NADPH oxidase
ALT: Alanine aminotransferase
PBS: Phosphate-buffered saline
TG: Triglyceride
GSH: Reduced glutathione
GSSG: Oxidized glutathione
TBARs: Thiobarbituric acid reactive substances
GPx: Glutathione peroxidase
SOD: Superoxide dismutase
H&E: Hematoxylin and eosin
TUNEL: Terminal deoxynucleotidyl transferase-mediated deoxyuridine triphosphate nick end labeling
*α*-SMA: *α*-Smooth muscle actin
TGF-*β*: Transforming growth factor *β*
ELISA: Enzyme-linked immunosorbent assay
COL1A1: Collagen 1A1
TIMP: Tissue inhibitor of matrix metalloproteinase
MMP: Matrix metalloproteinase
PDGF-R*β*: Platelet-derived growth factor receptor *β*
ROS: Reactive oxygen species
SDS-PAGE: Sodium dodecyl sulfate–polyacrylamide gel electrophoresis
PVDF: Polyvinylidene fluoride
ECL: Enhanced chemiluminescence
qRT-PCR: Quantitative real-time polymerase chain reaction
SD: Standard deviation.
